# AHL-Lactonase Producing *Psychrobacter* sp. From Palk Bay Sediment Mitigates Quorum Sensing-Mediated Virulence Production in Gram Negative Bacterial Pathogens

**DOI:** 10.3389/fmicb.2021.634593

**Published:** 2021-04-14

**Authors:** Issac Abraham Sybiya Vasantha Packiavathy, Arunachalam Kannappan, Sivaprakasam Thiyagarajan, Ramanathan Srinivasan, Danaraj Jeyapragash, John Bosco John Paul, Pazhanivel Velmurugan, Arumugam Veera Ravi

**Affiliations:** ^1^Department of Biotechnology, Karpagam Academy of Higher Education, Coimbatore, India; ^2^Department of Biotechnology, Alagappa University, Karaikudi, India; ^3^Department of Food Science and Technology, School of Agriculture and Biology, Shanghai Jiao Tong University, Shanghai, China; ^4^Fujian Provincial Key Laboratory of Agroecological Processing and Safety Monitoring, College of Life Sciences, Fujian Agriculture and Forestry University, Fuzhou, China; ^5^Key Laboratory of Crop Ecology and Molecular Physiology (Fujian Agriculture and Forestry University), Fujian Province University, Fuzhou, China; ^6^Department of Electronics and Communication Engineering, Karunya Institute of Technology and Sciences, Coimbatore, India; ^7^Centre for Materials Engineering and Regnerative Medicine, Bharath Institute of Higher Education and Research, Chennai, India

**Keywords:** AHL-lactonase, marine Palk Bay sediment, *Pseudomonas aeruginosa*, *Psychrobacter* sp., quorum quenching

## Abstract

Quorum sensing (QS) is a signaling mechanism governed by bacteria used to converse at inter- and intra-species levels through small self-produced chemicals called N-acylhomoserine lactones (AHLs). Through QS, bacteria regulate and organize the virulence factors’ production, including biofilm formation. AHLs can be degraded by an action called quorum quenching (QQ) and hence QQ strategy can effectively be employed to combat biofilm-associated bacterial pathogenesis. The present study aimed to identify novel bacterial species with QQ potential. Screening of Palk Bay marine sediment bacteria for QQ activity ended up with the identification of marine bacterial isolate 28 (MSB-28), which exhibited a profound QQ activity against QS biomarker strain *Chromobacterium violaceum* ATCC 12472. The isolate MSB-28 was identified as *Psychrobacter* sp. through 16S-rRNA sequencing. *Psychrobacter* sp. also demonstrated a pronounced activity in controlling the biofilm formation in different bacteria and biofilm-associated virulence factors’ production in *P. aeruginosa* PAO1. Solvent extraction, heat inactivation, and proteinase K treatment assays clearly evidence the enzymatic nature of the bioactive lead. Furthermore, AHL’s lactone ring cleavage was confirmed with experiments including ring closure assay and chromatographic analysis, and thus the AHL-lactonase enzyme production in *Psychrobacter* sp. To conclude, this is the first report stating the AHL-lactonase mediated QQ activity from marine sediment bacteria *Psychrobacter* sp. Future work deals with the characterization, purification, and mass cultivation of the purified protein and should pave the way to assessing the feasibility of the identified protein in controlling QS and biofilm-mediated multidrug resistant bacterial infections in mono or multi-species conditions.

## Introduction

Biofilms are a complex aggregation of mono or mixed species of microbial populations embedded on the biotic or abiotic surfaces by a self-produced extracellular polymeric matrix (Sharma et al., 2019). Biofilm forming microorganisms are responsible for a cluster of common hospital-acquired ailments including lung infection in patients with cystic fibrosis (CF), otitis media, periodontitis, burn wound infections caused by a variety of surgical implants, endocarditis, and urinary tract infections. National Institutes of Health (NIH) recommended that approximately 60% of human infections are the consequence of biofilm formation on human mucosa. Initial attachment and subsequent maturation of biofilm are the two important steps in host tissue colonization and subsequent persistent infections (Costerton et al., 1999). Bacteria living inside biofilms are habitually able to tolerate host immune responses and are distinctly highly resistant to different antibiotics (Costerton et al., 1999). Comparatively, this level will frequently surpass the maximum dosage level, and hence limit the efficient treatment available to control bacterial infections. The underlying mechanism of resistance is multi-factorial which includes restricted penetration, heterogeneous metabolic activity, and expression of certain genes conferring enhanced resistance to antibiotics. Hence, identification of such compounds with potential antibiofilm activity is imperative to combat the pathogenesis of these detrimental pathogens.

In most of the bacterial pathogens, quorum sensing (QS) mechanism regulates the biofilm formation and other virulence factors’ production, in order to establish pathogenesis in the host. This QS mechanism is also called the cell-to-cell communication system, as the bacteria communicate with each other at inter- and intra-species levels using small diffusible signal molecules called autoinducers (AIs). In Gram negative bacteria, N-Acyl Homoserine Lactone (AHL) is the prime AI responsible for QS (Papenfort and Bassler, 2016), which bind to their cognate receptor proteins that together activate the expression of QS-controlled genes (Whitehead et al., 2001). In *lux*I/R QS system, the LuxI family protein synthesizes AHL. The LuxR family protein binds with AHL and regulates the expression of many genes responsible for their coordinated behavior including motility, antibiotic biosynthesis, virulence factor production, and biofilm formation (Davies et al., 1998). Most importantly, the QS mechanism governs the biofilm formation in most of the bacterial pathogens; the QS inhibitory process termed as quorum quenching (QQ) has offered a novel target to control the biofilm-associated infections (Dong and Zhang, 2005; Costerton et al., 2007). Contrasting to antibiotics, QS inhibitors will not set bacteria under strong selective pressure to develop drug-resistance (Zhao et al., 2020). Besides QS, flagellar motility and exopolysaccharide (EPS) have also been found to be essential for bacterial aggregation and biofilm formation (Pratt and Kolter, 1998; Jaisi et al., 2007).

Secondary metabolites from marine organisms are considered an important source of biomolecules for drug discovery (Newman and Cragg, 2004; Borges and Simões, 2019). Bacteria associated with corals, sponges, and other organisms have been recognized as the factual producers of many bioactive compounds (Kelman et al., 2006; Freckelton et al., 2018). Though AHL degradation enzymes from bacterial isolates have been identified from different sources (Dong et al., 2002; Uroz et al., 2003; Ulrich, 2004; Hassan et al., 2016), the investigation of antibiofilm activity of bacteria, particularly from marine resources, are expected to act against antibiotic resistant bacterial pathogens (Huang et al., 2019; Zhou et al., 2019). Recently, a marine isolate showing a promising antibiofilm activity against *Pseudomonas aeruginosa* has been reported from red sea sediment (Rehman and Leiknes, 2018). Also, the literature evidenced these marine bacteria as one of the sources of secondary metabolites and other extracellular hydrolytic enzymes (Romano et al., 2017; Borges and Simões, 2019). Hence it is believed that bacteria from marine sediments (MSB) may also have the ability to produce several secondary metabolites that target bacterial QS mechanism. In light of this view, the present study aimed to isolate marine sediment bacteria that target the bacterial QS mechanism, and to divulge the mechanism of QS inhibition.

## Materials and Methods

### Bacterial Strains, Culture Media, and Conditions

Biomarker strains *Chromobacterium violaceum* (ATCC 12472) and Tn5 mini mutant CV026 were used to determine the QS inhibitory potential of marine bacterial isolates. The bacterial pathogens such as *Serratia marcescens* (FJ584421), *Pseudomonas aeruginosa* PAO1, *Vibrio parahaemolyticus* (ATCC 17802), and *V. vulnificus* (MTCC 1145) were the target pathogens used in this study. For ring closure assay, *Bacillus subtilis* ATCC 6633 was used as positive control. All these strains were cultured aerobically in Luria-Bertani (LB) broth (Hi Media, India) and incubated at their optimum temperature (30°C for *C. violaceum* and 37°C for rest of the strains). For the experiment, the OD of the pathogens was adjusted to 0.4 at OD_600nm_ from the overnight culture (1 × 10^8^ CFU/ml). As a standard cell suspension, 1% from the OD adjusted culture was used to inoculate the medium. As a negative control, Zobell marine broth (ZMB) was added to the wells of control sample.

### Primary Screening for Quorum Sensing Inhibitors – Soft Agar Overlay Assay

Marine bacterial strains were isolated from sediment samples collected from the Palk Bay coastal region using Zobell Marine Agar 2216 (Hi Media, India). All the isolates were patched on Zobell marine agar and allowed to grow for 24 h. Following incubation, the plates were overlaid with soft agar (0.7% agar) incorporated with 1% of *C. violaceum* (ATCC 12472) at standard cell suspension. Post incubation at 30°C for 18 h, the plates were observed for the violacein pigment inhibition (McLean et al., 2004). Bacterial isolate which showed efficient violacein inhibition alone was selected and further examined against the QS-mediated virulence inhibition in other bacterial pathogens.

### Preparation of MSB-28 Cell Free Supernatant

The cell free supernatant (CFS) of MSB-28 used in this experiment was prepared by inoculating 1% overnight culture of MSB-28 culture into 10 ml of ZMB. After 18 h of incubation the culture was centrifuged at 10,000 rpm for 10 min. After that, the culture supernatant was collected, filter sterilized (0.22 *μ*m syringe filter), and stored at 4°C for further use.

### Quantification of Violacein Production

The effect of MSB-28 on inhibition in violacein production was quantified by spectrophotometric analysis. Biosensor *C. violaceum* strain CV026 (OD_600nm_ = 0.1, 1% to the final volume of the growth medium) was added to the test tubes containing LB supplemented with 5 μM of N hexanoyl-l-homoserine lactone (C_6_-HSL, Sigma) alone as the control, and LB supplemented with C_6_-HSL and CFS of MSB-28 at various concentrations (5–20% v/v). The tubes were then incubated at 30°C for 18 h. After incubation, cell pellets of the control and treatment groups were collected by centrifugation at 8,000 rpm for 10 min. Equal volume of dimethyl sulfoxide was added to the pellet and vortexed vigorously (30 s), in order to precipitate the insoluble violacein. Post vortexing, the resultant mixture was centrifuged at 8,000 rpm for 10 min to separate the cells from the CFS, and then the CFS was measured at 585 nm spectrophotometrically (Hitachi U-2800, Japan; Choo et al., 2006).

### Strain Identification

Alkaline lysis method was used to isolate genomic DNA from MSB-28. Ribosomal 16S rRNA gene was amplified using eubacterial universal primers (forward primer 5'-AGAGTTTGATCCTGGCTCAG-3' and reverse primer 5'-ACGGCTACCTTGTTACGACTT-3'; Babu et al., 2009). The PCR conditions are as follows with initial denaturation at 94°C for 5 min followed by 30 cycles at 94°C for 30 s, 45°C for 30 s, and 72°C for 60 s with a Thermal Cycler (ABI). Sequencing of the 16S rRNA gene (about 1,500 bp) was done in Macrogen (Seoul, Korea). The CAP3 software was used to assemble, analyze, and to manually edit the raw sequences of 16S rRNA gene. Using BLAST analysis, the assembled 16S rRNA gene sequence of MSB-28 was then compared within the NCBI database (http://www/ncbi.nlm.nih.gov/).

### Biofilm Prevention Assays

#### Microtiter Plate Assay

The effect of CFS from *Psychrobacter* sp. against the biofilm forming ability of bacterial pathogens was examined using 24-well microtiter plate (MTP; Santhakumari et al., 2016). MTP wells containing 1 ml of growth medium were added with 1% standard cell suspension of the target pathogens and 5–20% v/v (50–200 μl) of *Psychrobacter* sp. CFS. Plates were kept at 37°C for 24 h without agitation to allow the development of biofilm. After incubation, the wells of MTP were rinsed thrice with distilled water to remove non-adherent cells and the wells with biofilms were stained with 0.4% w/v of crystal violet solution for 5 min. The stained biofilms were than solubilized by adding 1 ml of 95% ethanol, centrifuged at 7,000 rpm for 10 min. Then, the supernatant was measured at 650 nm under UV-visible spectrometer (HITACHI U-2800, Japan).

#### Confocal Laser Scanning Microscope Analysis

The target pathogens were allowed to form biofilms on glass slides (1 cm^2^) placed in 24-wells MTP containing growth medium supplemented with and without *Psychrobacter* sp. CFS (20% v/v). The experiment setup was incubated for 24 h at appropriate temperatures. Post incubation, biofilms in the glass slides were stained with 0.1% (w/v) of acridine orange and the stained biofilm cells were observed and imaged under a CLSM (Zeiss LSM 710, Carl Zeiss, Germany) at a magnification of 20x. The Z-stack analysis was done with the Zen 2009 software (Carl Zeiss, Germany). In addition, biofilm biovolume and average thickness of the biofilm formed in control and treated samples (z-stack images) were analyzed by COMSTAT software (kindly gifted by Dr. Claus Sternberg, DTU Systems Biology, Technical University of Denmark; Kannappan et al., 2019).

#### Growth Inhibition Assay

The growth inhibitory activity of CFS of *Psychrobacter* sp. was assessed by the well diffusion agar assay by following the guidelines of Clinical and Laboratory Standards Institute (2006). One hundred microliters of bacterial pathogens (0.5 Mc Farland turbidity, 1 × 10^8^ CFU/ml) was uniformly spread over the Muller-Hinton Agar (MHA, Hi Media, India) plate. Wells were punched out (4 mm in diameter) in the bacteria-spread agar plates and were loaded with various concentrations (50–200 μl) of the CFS of MSB-28. Wells with 200 μl of ZMB served as control. Following incubation at 37°C for 24 h, the zone of inhibition was observed and recorded from the MHA plates.

### *P. aeruginosa* PAO1 Assays

#### Extraction and Quantification of EPS

PAO1 was grown in test tubes in the presence and absence of *Psychrobacter* sp. CFS at 37°C and harvested at the late log phase. For EPS extraction, the cultures were centrifuged to remove the cell pellets, and the resulting CFS was syringe filtered through 20 μm nitrocellulose membrane filters. To the filtered CFS, three volumes of ice-cold absolute ethanol was added, which was then left undisturbed overnight at 2°C. The precipitated EPS was collected by centrifugation at 10,000 rpm for 20 min. The resulting precipitate was resuspended in milli-Q water and stored at −20°C until future use (Packiavathy et al., 2014).

EPS quantification was done by adding 1 ml of the EPS solution to 1 ml of cold 5% phenol and 5 ml of concentrated sulfuric acid. The reaction mixture turns to a red color. The intensity of the color is directly proportional to the EPS production. The EPS production was quantified spectrophotometrically at 490 nm (Hitachi U-2800, Japan; Dubois et al., 1956).

### Swimming and Swarming Motility Assays

PAO1 was grown in test tubes in the presence (20% v/v) and absence of *Psychrobacter* sp. CFS at 37°C and harvested at the late log phase. In the center of the swarming plates (1% peptone, 0.5% NaCl, 0.5% agar and 0.5% filter-sterilized d-glucose) and swimming agar plates (1% tryptone, 0.5% NaCl, and 0.3% agar), 5 microlitres of untreated control and *Psychrobacter* sp. CFS treated PAO1 cells were placed separately and were then kept for incubation at 37°C for 24 h (Déziel et al., 2001).

#### Rhamnolipid Assay

Overnight culture (50 μl) of PAO1 cells grown in the absence and in the presence of varying concentration of *Psychrobacter* sp. CFS (100 and 200 μl/ml) were added separately into the wells punctured on M8 medium containing petriplates supplemented with 1 mM MgSO_4_, 0.2% glucose, 0.5% casamino acids (CAA), 0.02% cetyl trimethyl ammonium bromide (CTAB), 0.0005% methylene blue, and 1.5% agar. The experimental setups were left undisturbed for 48 h at 37°C. Rhamonolipid production was observed by CTAB precipitation and dark blue hallow formation around the well (Caizza et al., 2005).

#### Drop Collapse Assay

The assay was carried out by following the protocol of Caizza et al. (2005). For this experiment, *Psychrobacter* sp. CFS treated and untreated PAO1 cells were harvested as described above. The cultures were centrifuged to remove the cell pellets. The supernatant was syringe filtered using a 0.22 μm membrane and serially diluted in distilled water. Equal volumes of the serially diluted supernatants were placed in circles on the backside of a 24 well plate lid and left undisturbed for bead formation. Samples that failed to form beads were defined to have drop collapse activity.

#### Biofilm Ring Assay

The assay was done by inoculating 1% of the standard cell suspension of PAO1 into glass tubes containing 1 ml of LB medium in the absence and presence (20% v/v) of *Psychrobacter* sp. CFS. After 24 h of incubation at 37°C, the cultures were removed from control and treated tubes and the biofilms were stained with 0.4% crystal violet and documented (Bordi et al., 2010).

### Effect of Solvent, Heat, and Proteinase K on QQ Activity of *Psychrobacter* sp.

#### Polarity Extractions

Equal volumes of *Psychrobacter* sp. CFS was mixed and vortexed thoroughly with solvents from highly non-polar to highly polar including benzene (B), petroleum ether (PE), chloroform (C), and ethyl acetate (EA). The CFS and solvent mixture were left overnight for phase separation. Solvent phase was collected, evaporated, and weighted. Further, the same was resuspended in a known volume of distilled water or dimethyl sulfoxide (DMSO) and stored at −20°C. The QS inhibitory potential of every organic phase was done by following the above said protocol using QS biomarker strain *C. violaceum* (ATCC 12472).

#### Heat Sensitivity

To confirm the nature of the quorum quencher molecule, the CFS of *Psychrobacter* sp. was incubated at different temperatures ranging from 0 to 80°C for 10 min. After heat treatment, the CFS of *Psychrobacter* sp. was assayed against *C. violaceum* CV026 with an exogenous supply of C6-HSL as mentioned above in violacein quantification assay. Following 24 h incubation, the cultures were observed for the violacein production.

#### Proteolytic Activity

A treatment group composed of 1 ml CFS of *Psychrobacter* sp. was added with 1 mg/ml of Proteinase K (Sigma Aldrich, United States). Nutrient broth (pH-7.4) with Proteinase K and CFS of *Psychrobacter* sp. without Proteinase K acted as positive control and negative control, respectively. All the three groups were incubated at 55°C for 18 h. Following incubation, the samples were assayed against QS biomarker strain *C. violaceum* (ATCC 12472) for violacein pigment production.

### Enzymatic Degradation of AHL by Marine Isolate *Psychrobacter* sp.

C_6_-HSL (5 mM) was incubated with 500 μl of *Psychrobacter* sp. CFS and incubated for 16 h. The preparative thin layer chromatography (TLC) was performed by spotting 4 μl of the supernatant treated C_6_-HSL. An equal volume of LB containing pure 5 mM C_6_-HSL was used as a negative (untreated) control. TLC was developed with methanol and water in the ratio of 60: 40 v/v. The developed TLC plates were overlaid with 3 ml of top agar (0.7%, LB agar) containing *C. violaceum* CV026 (OD_600nm_ = 0.1). Further, the plates were kept at 30°C for overnight or until adequate color development was achieved.

### Enzymatic Cleavage of Lactone Ring – Ring Closure Assay

As per the protocol described by Yates et al. (2002), an aliquot of the digestion media containing *Psychrobacter* sp. CFS (500 μl) treated C_6_-AHL (5 mM) was added with 10 mM HCl to lower the pH to the level of 2.0, and was kept under incubation for 48 h at 4°C. On the other hand, the digestion media containing 500 μl of PAO1 CFS treated C_6_-HSL (5 mM) acted as a negative control as it has acylase activity (Sio et al., 2006) and 500 μl of *B. subtilis* ATCC 6633 CFS treated C_6_-HSL (5 mM) acted as a positive control as it has lactonase activity (Pan et al., 2008); both were also subjected to acidification. After 48 h of incubation, HCl was left to evaporate, and the residue was suspended in 20 μl of LB broth. The lactone recyclization was induced by the acidification of the degradation mixture. After this treatment, the acidified mixture was spotted onto TLC and overlaid with 3 ml of top agar (0.7%, LB agar) containing *C. violaceum* (OD_600nm_ = 0.1). After overnight incubation, production of violacein by CV026 confirms the degradation activity of AHL lactonase (Carlier et al., 2003).

### Confirmation of AHL Degrading Activity by HPLC Analysis

AHL degrading activity was assessed by incubating 2 ml of 100 mM phosphate buffer containing 5 mM C_6_-HSL (Sigma-Aldrich, United States) with 2 mg of acetone precipitated *Psychrobacter* sp. CFS at 30°C for 10 h. To exclude any alkaline lactonolysis due to pH, the pH of the mobile phase was adjusted to 6.5 and the pH of cell free lysate was 7. Post incubation, 20 μl of the digestion mixture was subjected to HPLC analysis (Shimadzu, Kyoto, Japan) with a C18 reverse phase analytical silica column (250 × 4.6 mm; 5 μm). The fractions were eluted using methanol and water (v/v) as mobile phase in the ratio of 50:50 at a flow rate of 1 ml/min (Uroz et al., 2005).

### AHL Extraction and Detection

Many bacteria in the proteobacteria group have been reported to produce AHL. Consequently, the AHL produced could affect the biological activity of MSB-28. Hence, MSB-28 was examined for the synthesis of AHL molecules. MSB-28 was grown overnight in LB medium (100 ml) buffered with 50 mM 3-[N-morpholino] propanesulfonic acid (MOPS) to pH 5.5 to prevent spontaneous degradation of AHLs (Yates et al., 2002). After that, CFS was extracted twice with equal volumes of acidified ethyl acetate (0.1% v/v glacial acetic acid). The resulting extracts were concentrated to dryness under vacuum and resuspended in a minimal amount of sterile milli-Q water. The presence of AHL molecules present in the extracts was analyzed by spotting the AHL extracts on TLC plates (TLC aluminum sheets 20 cm × 20 cm, Merck, Germany). Synthetic C_6_-AHL was used as a control. TLC was developed with methanol and water at a ratio of 60: 40 v/v. Then the TLC plates were overlaid with 3 ml of top agar (0.7%, LB agar) seeded with *C. violaceum* CV026 (OD_600nm_ = 0.1). Further, the plates were kept at 30°C overnight or until the development of a violet color (Chen et al., 2013).

### Data Analysis

All the experiments were carried out in triplicate in three independent experiments. The results are expressed as means ± standard deviation (SD).

## Results

### Screening Marine Bacteria for Production of QS Inhibitors

Out of 106 isolates screened, four isolates were shown to inhibit the QS mediated pigment production in *C. violaceum* ATCC 12472. Among the four isolates, marine sediment bacteria MSB-28 showed a constant and profound inhibition of violacein production (Data not shown). Moreover, the zone of inhibition was found to be opaque and not transparent, which clearly indicates QS inhibitory activity of MSB-28 and not the growth inhibition (Supplementary Figure S1A).

### Strain Identification

The 16S rRNA gene sequence of MSB-28 was submitted to GenBank (NCBI; accession number: GU447235). Using BLAST analysis, the 16S rRNA gene sequence of MSB-28 was compared in the database and showed to have sequence similarity and identity with *Psychrobacter* sp. to a level of 98%.

### Quantification of Violacein Inhibition

The CFS of MSB-28 showed a concentration dependent violacein inhibition against the QS-biomarker strain *C. violaceum* CV026; however, 20% v/v concentration of MSB-28 showed a 98% drop in violacein production (Supplementary Figure S1B).

### Growth Inhibitory Activity of MSB-28

Antibacterial activity of MSB-28 CFS was assessed by well diffusion agar assay against the target pathogens such as PAO1, *S. marcescens*, *V. vulnificus*, and *V. parahaemolyticus*. Results revealed that none of the target pathogens displayed any growth inhibitory zones around the wells at tested concentrations (data not shown) and results are presented in Supplementary Table S1.

### Biofilm Prevention Assay

#### MTP Assay

The antibiofilm potential of *Psychrobacter* sp. CFS was assessed against the bacterial pathogens such as PAO1, *S. marcescens*, *V. vulnificus*, and *V. parahaemolyticus*. *Psychrobacter* sp. CFS exhibited concentration dependent activity against all tested pathogens and maximum inhibitions of 89, 71, 58, and 60%, were recorded at 20% v/v against PAO1, *S. marcescens*, *V. vulnificus*, and *V. parahaemolyticus*, respectively, and tabulated (Supplementary Table S1).

#### Microscopy Analysis – CLSM Observation

**Figure 1:**
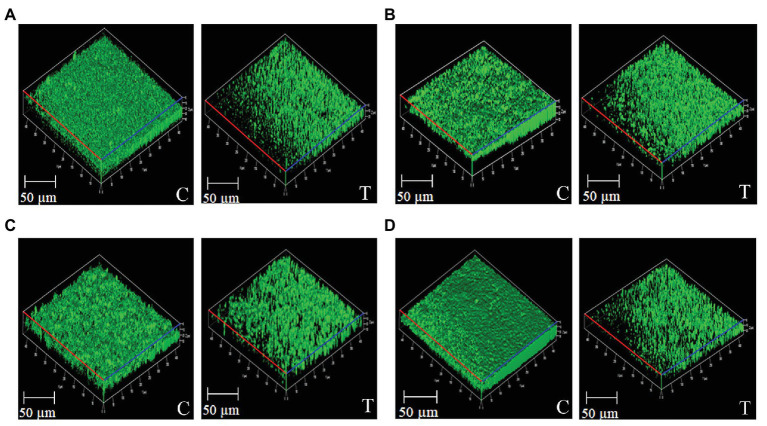
Confocal microscopy images of bacterial biofilms harbored with and without *Psychrobacter* sp. CFS (200 μl/ml). Images represent the untreated controls (insert-C) and *Psychrobacter* sp. treated (insert-T) biofilms of PAO1 **(A)**, *S. marcescens* **(B)**, *V. vulnificus* **(C)**, and *V. parahaemolyticus* **(D)**.

Consistent with the results of biofilm biomass quantification assay, CLSM analysis of the biofilms developed by the tested bacteria such as PAO1, *S. marcescens*, *V. vulnificus*, and *V. parahaemolyticus* displayed disintegrated biofilm structures in the presence of *Psychrobacter* sp. (Figure 1). Altogether, it is confirmed that the CFS of *Psychrobacter* sp. interferes with the biofilm formation of tested bacterial pathogens. COMSTAT software analysis was a quantitative method employed to analyze the biofilm formation of the tested pathogen using the raw LSM file (Kannappan et al., 2019). The COMSTAT analysis revealed that biofilm biovolume and average thickness of the tested pathogens were considerably reduced in the presence of *Psychrobacter* sp. CFS, which authenticated the antibiofilm potential of *Psychrobacter* sp. (Supplementary Table S2).

### Attenuation of QS Mechanisms in PAO1

#### Biosurfactant Inhibition-Rhamnolipid Assay

**Figure 2:**
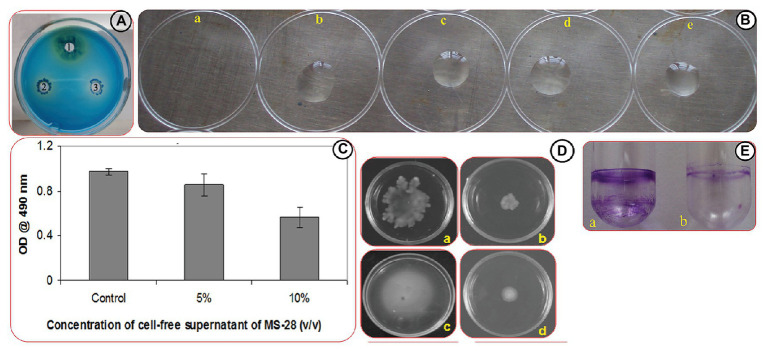
Effect of *Psychrobacter* sp. on virulence factors production in PAO1. **(A)** Rhamnolipd production in PAO1. Image represents rhamnolipid production in untreated control (1), and PAO1 treated with CFS of *Psychrobacter* sp. at of 100 (2) and 200 μl/ml (3) Concentrations. **(B)** Assessment of biosurfactant production through drop collapse assay. Images represent the activity of untreated PAO1 **(a)**, and PAO1 treated with CFS of *Psychrobacter* sp. at concentrations of 50–200 μl/ml **(b-e)**. **(C)** Quantitative analysis of EPS inhibition in PAO1 cells treated with and without *Psychrobacter* sp. CFS. Data represent the OD values of the EPS. Mean values represent the data of three independent experiments and SD are shown. **(D)** Effect of *Psychrobacter* sp. on the motility of PAO1. Swimming and swarming motilities of untreated control of PAO1 **(a,c)** and PAO1 treated with *Psychrobacter* sp. CFS (200 μg/ml; **b,d**), respectively. **(E)** Analysis of PAO1 biofilm formation in the absence **(a)** and presence **(b)** of *Psychrobacter* sp. through ring assay.

Interaction of CTAB with PAO1 surfactants leads to the precipitation of CTAB to form dark blue colonies surrounded by a white ring. In this study, the white halo surrounding the dark blue colonies around the well containing overnight culture of PAO1 clearly evidences the production of biosurfactant by PAO1, whereas wells containing cultures treated with *Psychrobacter* sp. CFS produced low levels of dark blue colonies when compared to control, indicating the reduced production of rhamnolipids (Figure 2A).

#### Biosurfactant Inhibition-Drop Collapse Assay

The overnight culture of PAO1 showed no visible bead formation, indicating the drop collapse capability of PAO1 by the production of biosurfactant. But the *Psychrobacter* sp. CFS treated PAO1 developed beads at all tested concentrations indicated the reduced production of biosurfactant (Figure 2B).

#### Inhibition of Biofilm EPS

EPS was extracted from both *Psychrobacter* sp. CFS treated and untreated cultures of PAO1. The obtained results revealed that CFS of *Psychrobacter* sp. inhibited the EPS production in PAO1 by 90% at 20% (v/v) concentration (Figure 2C).

### Swimming and Swarming Motility Inhibition Assays

As AHL-mediated QS regulates flagellar mediated motility of PAO1, the migration patterns of PAO1 in the presence of (20% v/v) of *Psychrobacter* sp. CFS were examined. As expected, the quorum quenching compound present in the *Psychrobacter* sp. had a great impact on the swarming and swimming migration patterns of PAO1 by displaying a significant reduction zone (Figure 2D).

#### Biofilm Ring Assay

The biofilm formation of PAO1 in borosilicate glass tubes (Figure 2E) was examined. For the untreated control tube, a thick layer of well-developed biofilm was easily stained by crystal violet. However, PAO1 cells treated with *Psychrobacter* sp. at a concentration of 20% (v/v) showed a remarked biofilm inhibition as evidenced by the thin biofilm ring.

### Effect of Solvent, Heat, and Proteinase K Treatment on QQ Activity of *Psychrobacter* sp.

**Figure 3:**
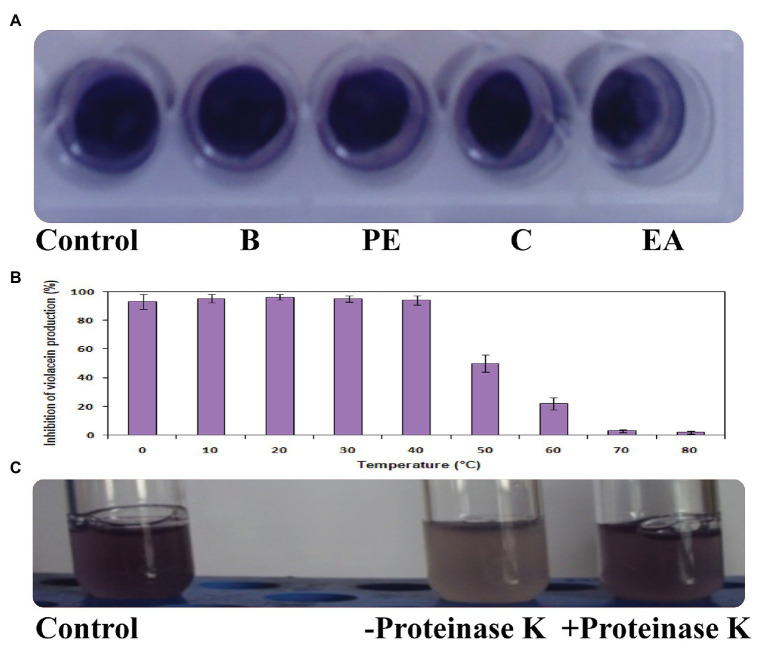
Effect of solvents **(A)**, heat treatment **(B)**, and proteinase K **(C)** on QQ activity of *Psychrobacter* sp.

The QQ activity was lost when the CFS of *Psychrobacter* sp. was extracted with different solvents as depicted in Figure 3A. In the heat-inactivation assay, *Psychrobacter* sp. CFS retained its potential QQ activity upon treatment with 0–40°C temperature for 10 min by showing the inhibition percentage of around 95%. The violacein inhibition percentage was reduced to 50% upon treatment with 50°C for 10 min. Similarly, around a 22% reduction was observed when subjected to heat treatment at 60°C. At higher temperatures (70 and 80°C), *Psychrobacter* sp. CFS completely lost its violacein inhibition activity (Figure 3B). The obtained results confirmed that the active lead present in the CFS of *Psychrobacter* sp. was heat sensitive. To confirm the enzymatic nature of QQ molecule, the CFS of *Psychrobacter* sp. was incubated with proteinase K (1 mg/ml). Pigment deficiency was observed with proteinase K treatment, whereas CFS of *Psychrobacter* sp. without proteolytic digestion retained its QQ activity (Figure 3C). These results clearly revealed the enzymatic nature of *Psychrobacter* sp. and its activity is possibly attributed to the presence of an AHL-degrading enzyme.

### AHL-Degrading Activity by *Psychrobacter* sp. Through TLC Analysis

**Figure 4:**
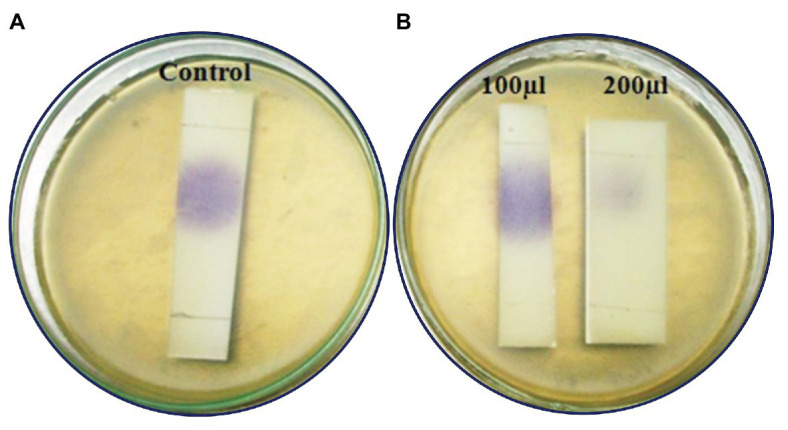
Analysis of C_6_HSL degradation by *Psychrobacter* sp. through TLC method. **(A)** C_6_HSL alone. **(B)** C_6_HSL treated with CFS of *Psychrobacter* sp. at different concentrations.

The inhibition of violacein pigment was observed in C_6_-HSL treated with *Psychrobacter* sp. CFS in TLC plate, which confirms the presence of AHL degrading enzymes in CFS of *Psychrobacter* sp. The inhibition was found to be dose dependent (Figure 4).

### Confirmation of AHL-Degrading Activity of MSB-28 by HPLC Analysis

**Figure 5:**
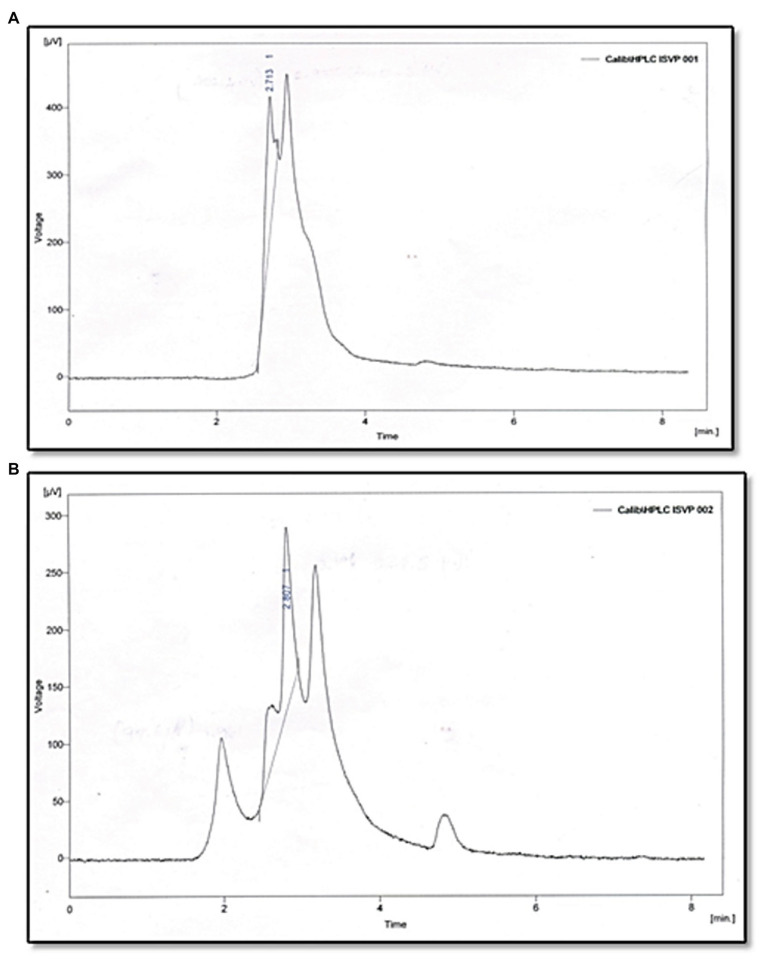
Analysis of C_6_HSL degradation by *Psychrobacter* sp. through HPLC. **(A)** C_6_HSL alone. **(B)** C_6_HSL treated with *Psychrobacter* sp.

The reaction products of C6-HSL digested with partially purified *Psychrobacter* sp. CFS as well as synthetic C6-HSL (without cell free lysate) were subjected to RP-HPLC analysis. The negative control C6-HSL displayed a major peak at the retention time of 2.7 min and a solvent peak at 3 min (Figure 5A). After 10 h incubation, the HPLC profile of the reaction mixture containing the partially purified cell free lysate with C6-HSL revealed a peak at a retention time of 1.9 min, which might correspond to hydrolyzed products of C6-HSL and a small peak corresponding to the remaining C6-HSL at 2.8 min, in addition to the solvent peak at 3.1 min (Figure 5B).

### Confirmation of Lactonase Activity-Lactonolysis Assay (Ring Closure Assay)

**Figure 6:**
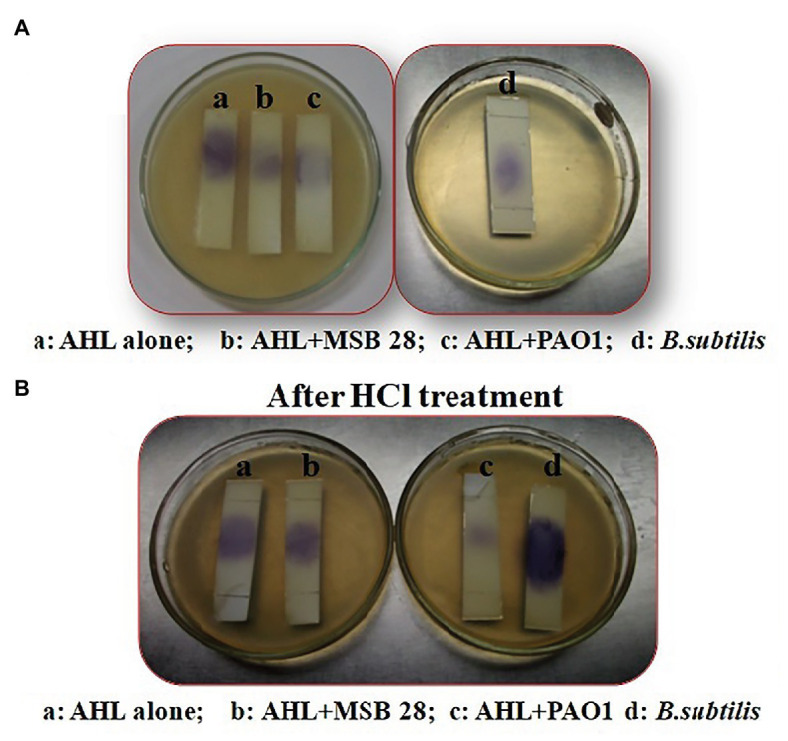
**(A)** Analysis of mechanism of action of *Psychrobacter* sp. by ring closure assay. **(B)** Activity after HCL treatment. C_6_HSL alone **(a)**, degradation pattern of C_6_HSL by CFS of *Psychrobacter* sp., **(b)** PAO1 **(c)**, and *B. subtilis* **(d)**.

Lactonase activity is defined as the cleavage of lactone ring in AHL (active QS molecule) to become N-acyl homoserine derivative (non-active QS signal). After AHL degradation, the AHL degradation media was treated with HCl to lower the pH to 2.0 in order to induce the lactone ring closure of the AHL and to restore the activity of AHL molecules. The biosensor *C. violaceum* CV026 restored its pigmentation upon incubation with acidified AHL degradation media containing C6-HSL treated with MSB-28 as well as the positive control *B. subtilis* (Figure 6). In contrast, no such activity was observed with supernatant from PAO1 which has already been reported to encompass acylase activity (Sio et al., 2006). Together, the results confirm the enzymatic compound responsible for QQ activity of *Psychrobacter* sp. could be an AHL lactonase and act upon the lactone ring of AHL.

### AHL Extraction and Detection

CV026 induced violacein production was not found in the TLC plate loaded with the extract from *Psychrobacter* sp. In contrast, violacein production was observed in the TLC plate loaded with synthetic C6-HSL (Supplementary Figure S2). The observed results lucidly revealed the nil production of AHL molecule(s) by the bacterium *Psychrobacter* sp. Hence, it is envisaged that the observed QQ activity with *Psychrobacter* sp. would not be plausibly interrupted by the AHL signaling mechanism as this bacteria lacks the ability to produce it.

## Discussion

The emergence of antimicrobial resistance is responsible for the failure of current antibiotics treatment on biofilm-based bacterial infections and has emphasized the urgent need for developing new alternate strategies. Bacterial QS mechanism seems to be an attractive target to develop an alternative therapeutic approach, as inhibition of QS hinders the virulence production, biofilm formation, and subsequent infection by many bacterial pathogens. Regardless, the marine bacterial organisms are being extensively investigated for their antimicrobial potentials; studies on their QQ potential are meager (Musthafa et al., 2011; Borges and Simões, 2019). Therefore, in this study, bacteria were isolated from Palk Bay sediment samples and screened against QS biomarker strain *C. violaceum*. Except the CFS of marine isolate MSB-28, none of the isolates showed pronounced QQ activity against *C. violaceum* 12472. Similar reports were made by Shepherd and Lindow (2009) in which QQ activity was observed in CFS of *P. syringae* B728a which enabled the bacteria to degrade QS signals and thus block the expression of QS-regulated traits. The isolate showing profound QQ activity was found to be *Psychrobacter* sp. and belongs to Proteobacteria. These results corroborate well with earlier studies, where the bacteria isolated from a marine environment with QQ potential were identified as Proteobacteria (Tan et al., 2015; Torres et al., 2016; Rehman and Leiknes, 2018).

QS governs the biofilm formation and maturation in several bacterial species. The marine bacterial isolate *Psychrobacter* sp. exhibited biofilm inhibition against various Gram-negative pathogens such as PAO1, *S. marcescens*, *V. vulnificus*, and *V. parahaemolyticus* at 20% v/v without growth retardation (Supplementary Table S1). The degree of variation in the QQ activity of *Psychrobacter* sp. could be due to the involvement of AHLs produced by the target bacteria with varied lengths of acyl side-chain. Flagella and pili aid to initiate the biofilm formation by reversible and irreversible attachment followed by microcolony formation (Shi and Sun, 2002). Hence, any interference in their expression by the metabolites of *Psychrobacter* sp. would result in the failure of biofilm formation. Development of distinctive biofilm architecture is the most important stage in biofilm formation (Kannappan et al., 2017). The attained results of CLSM analysis suggest that biofilm formation of the target pathogens was inhibited at the early stages of biofilm development. Moreover, the result of COMSTAT analysis ascertained the biofilm quantification assay. Altogether, these results suggest that the QQ agent present in *Psychrobacter* sp. might possibly interrupt the biofilm development without any negative effect on the bacterial growth. Bacteria are known to secrete antibacterial compounds. The growth analysis of pathogens clearly portrayed that the CFS of *Psychrobacter* sp. had no antibacterial activity towards the target pathogens. QS inhibition without any growth reduction is considered as the best alternative strategy to control the virulence factors’ production and pathogenesis of bacterial pathogens, and leaves no scope for the development of antibiotics resistance (Rasmussen and Givskov, 2006). In this light, CFS of *Psychrobacter* sp. showed a profound QQ activity without any growth inhibition against the representative Gram-negative pathogens, which holds great clinical significance.

We also examined the QQ ability of *Psychrobacter* sp. to control other virulence factors associated with biofilms produced by PAO1. Production of EPS is known to maintain the biofilm architecture, and also correlates with an increased resistance of the biofilm-residing cells to biocides and host immune response (Kannappan et al., 2017). Hence, inhibiting EPS secretion by marine bacterium *Psychrobacter* sp. would loosen the biofilm architecture; thus, it is possible to reintroduce the use of antibiotics in treating biofilm cells along with active leads produced by *Psychrobacter* sp. In *P. aeruginosa*, several QS regulated phenotypic behaviors have been reported to be a part of the biofilm formation (Fong et al., 2019). In this study, treatment of *Psychrobacter* sp. would result in the reduced production of rhamnolipid; an important factor enhances the swarming motility by reducing the surface tension. In contrast, deficiency in surfactant production alters the swarming migration pattern and the altered bacteria would fail to colonize over the surface. The possible mode of action of the *Psychrobacter* sp. to block biofilm development is interfering either with C4-HSL signaling pathway accountable for surfactant production and swarming motility or blockage of 3-oxo-C_12_ HSL signals which have a direct control over biofilm formation. As signal-mediated QS regulates the virulence factors’ production and biofilm formation, a remarkable reduction in biofilm formation and associated behaviors by *Psychrobacter* sp. might result from an effective hindrance of signal molecules by the secondary metabolites from *Psychrobacter* sp. Consistent with this result, a marine isolate VG-12 from red sea sediment inhibited the biofilm formation of PAO1 *via* QS signal degradation (Rehman and Leiknes, 2018).

The CFS of *Psychrobacter* sp. that lost its QQ activity upon being subjected to solvent extraction (Figure 3A), heat (Figure 3B), and Proteinase k (Figure 3C) treatments indicate that bioactive lead produced by *Psychrobacter* sp. is enzymatic in nature. Moreover, it is suggested that these QQ enzymes are heat sensitive. Hence, it is speculated that the loss of AHLs signaling was either because of AHL acylase or AHL lactonase activity. It is known that QQ bacteria able to degrade small-chain AHLs can also degrade medium and long-chain AHLs (Tan et al., 2015; Torres et al., 2016; Rehman and Leiknes, 2018). Hence, it is envisaged that investigation of QQ bacteria should have a focal point on identifying bacteria that targets small-chain AHLs, as recommended previously (Rehman and Leiknes, 2018). Interestingly, in this study the marine isolate was able to degrade the external C6-AHLs (Figure 5). AHL lactonases and AHL acylases are the best-known examples of AHL degrading so far reported and studied. Though the activity of AHL-acylases on short-chain AHLs remains unclear (Shepherd and Lindow, 2009; Czajkowski et al., 2011), it would be factual if the degraded C6-AHLs will be restored after acidification. In the present study, the degradation and restoration of short chain C6-AHLs suggest that the observed QQ activity of *Psychrobacter* sp. in attenuating the QS-mediated biofilm formation by bacterial pathogens such as *S. marcescens*, *P. aeruginosa*, *V. parahemolyticus*, and *V. vulnificus* is possibly due to the presence of an AHL lactonase. Altogether, the obtained results from the present study evidence that the AHL lactonase produced by the marine bacterium *Psychrobacter* sp. is heat-liable and active against different AHLs produced by other pathogens. Moreover, this bacterium was not found to produce any AHL signal molecule and hence could be used as a potent source for AHL degrading lactonase enzyme. For the first time, the present report divulged the quorum quenching lactonase enzymes production from *Psychrobacter* sp.

## Conclusion

Quorum quenching is of great concern in controlling infectious pathogens without interfering with growth, thus avoiding the selection pressure that often results in the emergence of resistance strains. In this study, we found marine sediment bacteria with QQ potential, identified as *Psychrobacter* sp., that is able to degrade AHLs and thereby inhibit the QS mechanism and biofilm formation of diverse bacterial pathogens. Moreover, lactonolysis and chromatograpic analysis revealed the presence of AHL-lactonase in the CFS of *Psychrobacter* sp. Thus, the attained results emphasize that the QQ activity of *Psychrobacter* sp. could potentially be used as a biocontrol agent to combat multidrug resistant bacterial infections caused by Gram-negative human as well aquatic pathogenic bacteria.

## Data Availability Statement

The datasets presented in this study can be found in online repositories. The names of the repository/repositories and accession number(s) can be found in the article/Supplementary Material.

## Author Contributions

IP: conceptualization, performed the experiments, data analysis, and writing – original draft. AK and ST: performed the experiments, data analysis, and writing and reviewing original draft. RS: data analysis, and writing and reviewing original draft. DJ and JP: data analysis. PV: data validation. AR: conceptualization, supervision, data validation, and writing – original draft. All authors contributed to the article and approved the submitted version.

### Conflict of Interest

The authors declare that the research was conducted in the absence of any commercial or financial relationships that could be construed as a potential conflict of interest.

## Supplementary Material

Supplementary Figure S1 **(A)** Assessment of QQ activity of MSB-28 using *C. violaceum* 12472 by soft agar overlay assay. **(B)** Quantitative assessment of violacein inhibition in CV026 by CFS of MSB-28 at increasing concentrations. Mean values represent the data of three independent experiments and SD are shown.

Supplementary Figure S2 Detection of AHL produced by *Psychrobacter* sp. *via* the biosensor CV026. **(a)** Natural C_6_-HSL as positive control, and **(b)** extract from the spent culture supernatant of *Psychrobacter* sp.
