# Supplementary figures and images for: AHL-Lactonase Producing Psychrobacter sp. From Palk Bay Sediment Mitigates Quorum Sensing-Mediated Virulence Production in Gram Negative Bacterial Pathogens
Source: Front Microbiol. 2021 Apr 14;12:634593. doi: 10.3389/fmicb.2021.634593 (PMC8079732; doi:10.3389/fmicb.2021.634593)

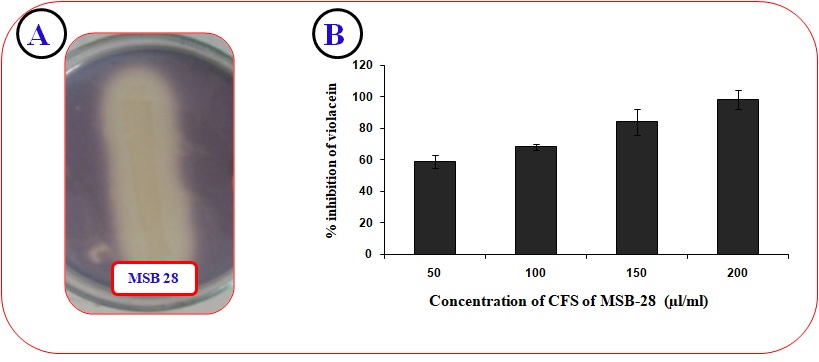

Supplement: Supplementary Figure S1 — (A) Assessment of QQ activity of MSB-28 using C. violaceum 12472 by soft agar overlay assay. (B) Quantitative assessment of violacein inhibition in CV026 by CFS of MSB-28 at increasing concentrations. Mean values represent the data of three independent experiments and SD are shown. [file Image_1.jpg]

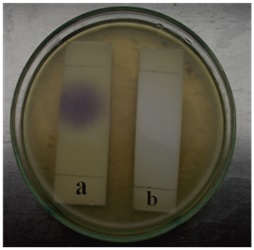

Supplement: Supplementary Figure S2 — Detection of AHL produced by Psychrobacter sp. via the biosensor CV026. (a) Natural C6-HSL as positive control, and (b) extract from the spent culture supernatant of Psychrobacter sp. [file Image_2.jpg]
