# Supplementary material for: AHL-Lactonase Producing Psychrobacter sp. From Palk Bay Sediment Mitigates Quorum Sensing-Mediated Virulence Production in Gram Negative Bacterial Pathogens
Source: Front Microbiol. 2021 Apr 14;12:634593. doi: 10.3389/fmicb.2021.634593 (PMC8079732; doi:10.3389/fmicb.2021.634593)
Supplement: Supplementary file 3 [file Table_1.DOC]

**Table 1:** Antibiofilm activity of *Psychrobacter* sp. against Gram-negative bacterial pathogens. At the tested biofilm inhibitory concentration (200 µl/ml), *Psychrobacter* sp. showed no growth inhibition on the test pathogens, but only the biofilm inhibition.

| **Bacterial strain** | **Concentration of the CFS of**  ***Psychrobacter* sp.** (**µl/ml)** | **Growth inhibition** | **Biofilm inhibition**  **(%)** | **SD** |
| --- | --- | --- | --- | --- |
| PAO1 | 200 | Nil | 89.21 | ±2.5 |
| *Serratia marcescens* | 200 | Nil | 70.56 | ±2.7 |
| *Vibrio vulnificus* | 200 | Nil | 58.37 | ±1.8 |
| *Vibrio parahaemolyticus* | 200 | Nil | 59.86 | ±1.1 |
