# Supplementary material for: AHL-Lactonase Producing Psychrobacter sp. From Palk Bay Sediment Mitigates Quorum Sensing-Mediated Virulence Production in Gram Negative Bacterial Pathogens
Source: Front Microbiol. 2021 Apr 14;12:634593. doi: 10.3389/fmicb.2021.634593 (PMC8079732; doi:10.3389/fmicb.2021.634593)
Supplement: Supplementary file 4 [file Table_2.DOC]

**Table 2.** COMSTAT analysis of biovolume and average thickness of the biofilm formed by the tested pathogen in the presence and absence of *Psychrobacter* sp. CFS.

|  | **Biofilm biovolume (µm3 /µm2)** | | **Average thickness (µm)** | |
| --- | --- | --- | --- | --- |
|  | **Control** | **Treated** | **Control** | **Treated** |
| **PAO1** | 85.35±2.17 | 38.21±1.18 | 80.66±3.13 | 28.37±0.81 |
| ***S. marcescen s*** | 78.62±1.23 | 44.69±0.67 | 72.53±3.20 | 35.44±2.47 |
| ***V. vulnificus*** | 70.41±1.55 | 46.22±2.01 | 68.31±1.93 | 32.79±2.18 |
| ***V. parahaemolyticus*** | 98.55±0.74 | 39.88±2.49 | 95.93±4.01 | 31.57±1.19 |
